# The evolutionary differentiation of two histone H2A.Z variants in chordates (H2A.Z-1 and H2A.Z-2) is mediated by a stepwise mutation process that affects three amino acid residues

**DOI:** 10.1186/1471-2148-9-31

**Published:** 2009-02-04

**Authors:** José M Eirín-López, Rodrigo González-Romero, Deanna Dryhurst, Toyotaka Ishibashi, Juan Ausió

**Affiliations:** 1 Departamento de Biología Celular y Molecular, Universidade da Coruña, E15071 A Coruña, Spain; 2 Department of Biochemistry and Microbiology, University of Victoria, V8W 3P6 Victoria, BC, Canada

## Abstract

**Background:**

The histone H2A family encompasses the greatest number of core histone variants of which the replacement variant H2A.Z is currently one of the most heavily studied. No clear mechanism for the functional variability that H2A.Z imparts to chromatin has yet been proposed. While most of the past studies have referred to H2A.Z generically as a single protein, in vertebrates it is a mixture of two protein forms H2A.Z-1 (previously H2A.Z) and H2A.Z-2 (previously H2A.F/Z or H2A.V) that differ by three amino acids.

**Results:**

We have performed an extensive study on the long-term evolution of H2A.Z across metazoans with special emphasis on the possible selective mechanisms responsible for the differentiation between H2A.Z-1 and H2A.Z-2. Our results reveal a common origin of both forms early in chordate evolution. The evolutionary process responsible for the differentiation involves refined stepwise mutation change within the codons of the three differential residues. This eventually led to differences in the intensity of the selective constraints acting upon the different H2A.Z forms in vertebrates.

**Conclusion:**

The results presented in this work definitively reveal that the existence of H2A.Z-1 and H2A.Z-2 is not a whim of random genetic drift. Our analyses demonstrate that H2A.Z-2 is not only subject to a strong purifying selection but it is significantly more evolutionarily constrained than H2A.Z-1. Whether or not the evolutionary drift between H2A.Z-1 and H2A.Z-2 has resulted in a functional diversification of these proteins awaits further research. Nevertheless, the present work suggests that in the process of their differently constrained evolutionary pathways, these two forms may have acquired new or complementary functions.

## Background

In eurkaryotic organisms, DNA is found associated with histone proteins constituting a nucleoprotein complex called chromatin. Approximately 146 base pairs of DNA wrap around a core histone octamer to form a nucleosome which is the basic subunit of chromatin. This nucleoprotein complex allows for the high extent of compaction of genomic DNA within the cell nucleus and provides the support on which most DNA metabolic processes take place [1]. There are five histone families which can be classified into core histones (H2A, H2B, H3, and H4) and linker histones (H1) according to structural and functional features. The histone H1 and H2A families show the most diversity of isoforms that have dedicated functions in many cellular processes including organization of chromatin structure in somatic and germinal cells, gene transcription, DNA replication, and DNA repair among others [2-9].

The histone H2A family contains the greatest number of variants among the core histones, some of which are essential for the maintenance of genome integrity and viability such as H2A.Z and H2A.X [2,10,11]. At present, H2A.Z is one of the most heavily studied histone variants and it has been ascribed multiple functions that may differ among species. In yeast, H2A.Z (Htz1) is present at active and inactive gene promoters in euchromatin, it is depleted at the silenced subtelomeric heterochromatin and it is enriched at the boundaries between euchromatin and heterochromatin [12]. Although studies concerning the function of H2A.Z in mammalian cells have always yielded results that seem difficult to reconcile, a growing body of evidence suggests that H2A.Z is present at gene promoters and that in an acetylated form, its presence correlates with gene expression [13,14]. However, it appears there are also at least two populations of H2A.Z present in heterochromatin. Greaves and colleagues show that H2A.Z is a feature of pericentric heterochromatin and contributes to the structure of the centromere [15]. Another fraction of H2A.Z stains the length of the inactive X chromosome though intriguingly this fraction can be distinguished by monoubiquitination at K120, K121 or K125 [16].

Analysis of the H2A.Z-containing nucleosome has also yielded conflicting results [3,17,18]. The crystal structure of this nucleosome initially suggested a subtle destabilization between the H2A.Z-H2B dimer and the H3-H4 tetramer [19]. However, FRET and analytical ultracentrifuge analysis using native H2A.Z have indicated that the H2A.Z nucleosome is in fact slightly more stable than the canonical H2A nucleosome [20,21]. Also, when H2A.Z is present in nucleosome arrays it facilitates the formation of the 30 nm chromatin fiber [22]. The recent study by Sarcinella and colleagues showed that it is a monoubiquitinated form of H2A.Z that is present on the inactive X chromosome [16]. This study is a clear demonstration that a post-translational modification has the potential to define a subpopulation of H2A.Z. Indeed, a similar situation can be seen with H2A.Z N-terminal acetylation and active gene transcription [14]. The difference in PTMs could reflect the different functional constraints of the H2A.Z-containing mono-nucleosomes in the *in vivo *setting. Conversely, the tendency to fold the chromatin fiber in arrays consisting of contiguous H2A.Z-containing nucleosomes may account for the presence of this variant in physiologically relevant situations such as that found in association with PcG proteins in the polycomb genes [23] or at the flanking sites of the insulator protein CTCF [24].

Mass spectrometry analysis showed that purified H2A.Z consists of an almost equimolar amount of two similar yet distinct proteins that differ by three amino acids [25] which are labeled here as H2A.Z-1 and H2A.Z-2. In the present work we have explored the long-term evolutionary pathway of H2A.Z-1 and H2A.Z-2 differentiation across metazoans and have analyzed the possible selective mechanisms and the constraints responsible for the potential functionalization of both variants. Our results have important implications for histone evolution and function as they show for the first time that H2A.Z-1 and H2A.Z-2 represent two very closely related variants that share a common evolutionary origin early in chordate evolution. Furthermore, our results show that the evolutionary constraints leading to the differentiation of both variants are primarily acting at the nucleotide level. This involves a refined process of stepwise mutation change within the codons of their three characteristic amino acid residues. Finally, we show that H2A.Z-2 is more tightly controlled (constrained) by selection than H2A.Z-1.

## Results and Discussion

### The phylogenetic context of H2A histone variants

**Figure 1:**
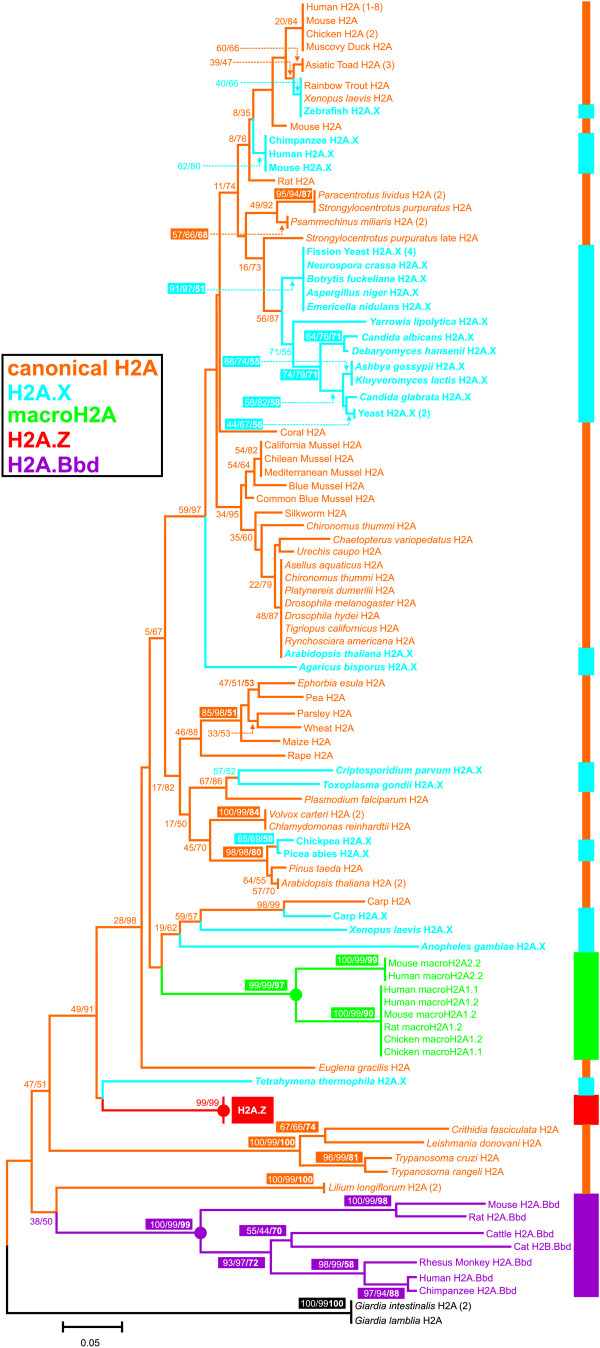
**Phylogenetic relationships among histone H2A proteins**. The reconstruction was carried out by calculating the evolutionary amino acid *p*-distances from the H2A sequences of all the organisms analyzed (see Additional file 1). Histone H2A types are indicated on the right near the species names, including canonical H2A proteins (orange), and the variants H2A.X (blue), macro H2A (green), the H2A.Z fraction (red) and H2A.Bbd (purple). The root of the tree is labeled in black. Green, purple and red circles denote the monophyletic origin for the corresponding group of variants. Numbers for interior nodes indicate BP/CP confidence values. Numbers in colored boxes and in boldface account for the bootstrap values obtained in the reconstruction of the maximum parsimony trees using all the informative positions in the alignment. Confidence values were based on 1000 replications and are only shown if at least one of the values is >50%.

The phylogeny including the sequences for all H2A histone variants known to date is shown in Fig. 1 (see Additional file 1 for a complete list of the sequences used and their accession numbers). It can be seen that some of these variants including histone H2A.Bbd [5,26], macroH2A [4,27], and the group comprising H2A.Z variants [28] have a monophyletic origin. Histone H2A.X shows a recurrent differentiation across evolution, implying that it has had multiple evolutionary origins as previously reported [2,11]. The single evolutionary origin of the remaining H2A variants is strongly supported by high levels of confidence in topology. Their distinctive identity with respect to the canonical H2A histones has been maintained since this origin. The clustering pattern in the tree is consistent with the functional diversification and differentiation of the histone subtypes based on a birth-and-death model of evolution [28], ruling out a major effect of a concerted evolution mechanism.

**Figure 2:**
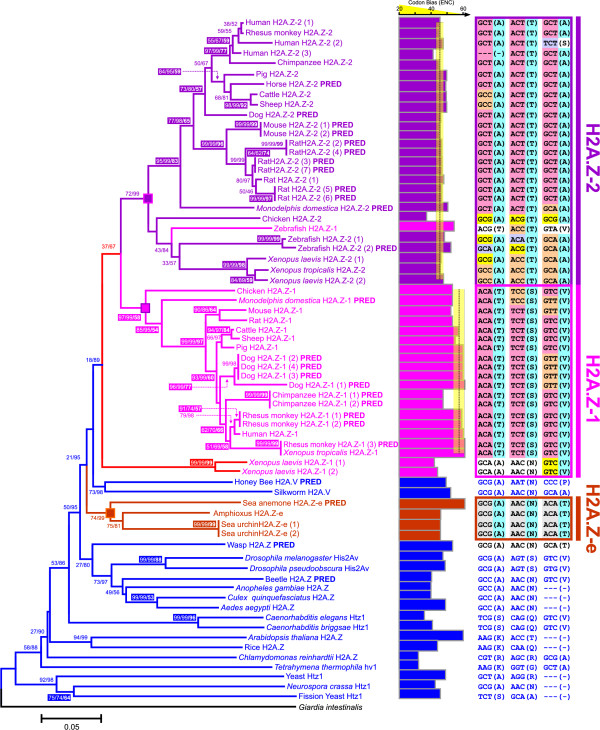
**Phylogenetic neighbor-joining tree showing the progressive specialization of H2A.Z-1 and H2A.Z-2 genes (indicated in Additional file **2**) across chordates after estimating the nucleotide *p*-distances**. Histone H2A.Z-1 variants are indicated in pink, H2A.Z-2 variants are indicated in purple and H2A.Z-e sequences from early chordates are indicated in brown. Histone variants from protostomes, plants and fungi/protists are indicated in blue, while the root of the tree is labeled in black. Pink, purple and red boxes in interior branches denote the monophyletic origin for each group. BP, CP and bootstrap confidence values for the maximum parsimony trees are indicated as in Fig. 1. Variant sequences predicted from databases and draft genomes data are indicated by PRED near the species name. The colored bar chart at the right indicates the codon usage bias for the corresponding sequence within each variant, estimated as the effective number of codons (ENC) and indicating the average (lines) and standard deviation (yellow box) for H2A.Z-1 and H2A.Z-2 sequences. The composition of the triresidue (amino acids and encoding triplets) is detailed in the right margin of the topology for each of the sequences analyzed and in different colors in case of variation in the codons.

As mentioned earlier, purified H2A.Z consists of two different forms. While one component of this mixture is referred to as H2A.Z, the other component is represented by a protein which was initially labeled as H2A.F/Z or H2A.V, which in humans differs at three residues from H2A.Z as follows: H2A.Z-Thr → H2A.V-Ala (pos. 15, Ala in canonical H2A), H2A.Z-Ser → H2A.V-Thr (pos. 39, Lys in canonical H2A), H2A.Z-Val → H2A.V-Ala (pos. 128, Lys in canonical H2A). Considering the small amino acid differences existing among H2A.Z, H2A.F and H2A.V and given that any of their protein sequences is closer to H2A.Z than to any other histone H2A variant, we will refer to them here as H2A.Z-1 (H2A.Z), H2A.Z-2 (H2A.V/F) and H2A.Z-e. The latter corresponds to histone H2A.Z from organisms preceding and including the early chordates, which is present previous to the differentiation between H2A.Z-1 and H2A.Z-2 that takes place in vertebrates (see Fig. 2 for details). Histone H2A.Z-2 has been mainly identified from cDNA libraries and during the annotation process of different genomes yet exhaustive studies on this variant are still lacking. Our preliminary biochemical results (manuscript in preparation) reveal the co-existence of both H2A.Z-1 and H2A.Z-2 mRNAs in chicken as well as in mammalian cells, showing significant differences in their expression levels across different tissues. Despite being preliminary, these results may lend support to the notion that H2A.Z-1 and H2A.Z-2 are functionally distinct and that they could act independently to fulfill the different roles played by the histone H2A.Z fraction in chromatin structure and function.

**Figure 3:**
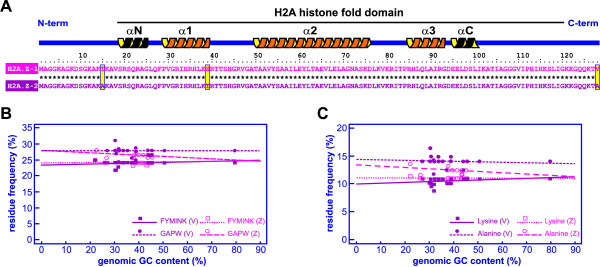
**Amino acid variation between H2A.Z-1 and H2A.Z-2 variants and selection for GC-rich and GC-poor amino acids**. **A **Protein alignment of H2A.Z-1 (pink) and H2A.Z-2 (purple) from human showing the three amino acid differences between both sequences constituting the triresidue (yellow boxes). Asterisks denote matching positions in the alignment and the secondary structure for both variants is represented above the alignment indicating the N-terminal region, the histone-fold domain and the C-terminal region. **B **Relationship between GC-content and the frequencies of GC-rich (GAPW) and GC-poor (FYMINK) amino acid classes, and (**C**) between GC content and the frequencies of alanine and lysine residues in H2A.Z-1 (pink) and H2A.Z-2 (purple) variants.

We have performed extensive DNA and protein database mining based on the differences exhibited by H2A.Z-1 (H2A.Z) and H2A.Z-2 (H2A.V) in three amino acid residues (referred to as triresidue hereafter, see Fig. 3A). The analysis made use of all the H2A.Z-2 sequences available, including those whose functionality has been assessed, predicted and even sequences in silico isolated from draft genomes. We obtained 21 H2A.Z-1 and 26 H2A.Z-2 sequences from chordates, which in many instances had been incorrectly annotated in the databases (see Additional file 2 for complete details). Inclusion of all H2A.Z-1 and H2A.Z-2 variants in the H2A protein phylogeny (Additional file 3) shows that their sequences overlap extensively without any evident functional clustering pattern or high confidence levels for the groups defined by the topology. This suggests that the differentiation between H2A.Z-1 and H2A.Z-2 has occurred beyond the triresidue difference that is observed in vertebrates.

Therefore, a phylogeny based on the complete nucleotide coding regions was reconstructed in order to further investigate the mechanisms of differentiation between H2A.Z-1 and H2A.Z-2. In contrast to the protein phylogeny, this defines a clear pattern between H2A.Z-1 and H2A.Z-2 in vertebrates (Fig. 2), with a monophyletic origin which is already independent from that of H2A.Z-e genes in early chordates, protostomes and fungi.

Two exceptions to this clustering pattern were detected. In the first instance, histones H2A.Z-1 and H2A.Z-2 from zebrafish are included in the same group. This could be due to a recent gene duplication within a short period of time (not enough time elapsed to allow for the accumulation of nucleotide substitutions). Under such circumstances, the presence of gene conversion would be unlikely because H2A.Z-1 and H2A.Z-2 genes are located on different chromosomes in zebrafish. Another exception was observed for the H2A.Z-1 genes from *X. laevis *which fall into an independent group outside both the H2A.Z-1 and H2A.Z-2 clusters. Given that the H2A.Z-1 gene from *X. tropicalis *and those of *X. laevis *and *X. tropicalis *H2A.Z-2 fall within their corresponding groups in the tree, the independent position of *X. laevis *H2A.Z-1 could be due to the extensive nucleotide variation exhibited among H2A genes.

The phylogeny from Fig. 2 represents a very useful tool in defining the identity of vertebrate H2A variants as either H2A.Z-1 or H2A.Z-2 on the basis of their nucleotide sequences. Indeed, the current topology allowed us to define 24 sequences previously annotated as H2A either as H2A.Z-1 or H2A.Z-2 following their position on the tree. Furthermore, 20 of those sequences were previously annotated as unknown in the databases, and thus the present analysis helped to reveal their true identity. In two instances (chicken and rhesus monkey) these sequences were wrongly defined as H2A.Z-2 in the databases, and our analyses unveiled their H2A.Z-1 identity (see Additional file 2). Such a clear differentiation between both histone H2A.Z forms using nucleotide phylogeny suggests not only a common phylogenetic origin early in metazoan evolution, but also the presence of a process of functional differentiation similar to that described for other histone multigene families [28-31]. However, the differentiation among other histone family members commonly encompasses extensive synonymous divergence under strong purifying selection at the protein level [28,29,32-34]. In contrast, the differentiation between H2A.Z-1 and H2A.Z-2 seems to primarily involve variation at the nucleotide level which in vertebrates resulted in a subtle protein differentiation encompassing three different amino acid residues. Such structural amino acid refinement represents a new mechanism among those previously known in histone diversification and is further investigated below.

### Protein and nucleotide variation in H2A.Z-1 and H2A.Z-2 variants

The amino acid sequence alignments shown in Fig. 3A and Additional file 4 highlight the high extent of protein similarity between H2A.Z-1 and H2A.Z-2. This is especially evident when comparing the sequences in the same species, as in the case of human H2A.Z-1 and H2A.Z-2 (Fig. 3A). The alignment reveals the presence in vertebrates of only three residue differences (triresidue) defined as: Thr → Ala (pos. 15, N-terminal region), Ser → Thr (pos. 39, central globular region), and Val → Ala (pos.128, C-terminal region). The amino acid residues involved in the acidic patch of H2A [35] that are presumably involved in interchromosomal contacts within the chromatin fibre [36] are maintained in both H2A.Z-1 and H2A.Z-2 in contrast to what has been observed in other H2A variants such as H2ABbd [26,37]. Although the second amino acid of the triresidue falls within the a-helix 1 domain of the histone fold [38], a computer analysis using molecular dynamic simulation (see materials and methods) indicated that such variation has no structural implications for secondary or tertiary structure of these histone variants (Additional file 4).

Given the highly charged nature of histones and their involvement in protein-DNA interactions that modulate chromatin dynamics, the electrostatic interaction properties of the different H2A variants were analyzed. Electrostatic potentials and the corresponding similarity indices were calculated for all H2A.Z-1 and H2A.Z-2 proteins listed in Additional file 2, allowing us to calculate the electrostatic distances between proteins (Additional file 5). As expected from Additional file 4, this analysis showed that the differentiation process between H2A.Z-1 and H2A.Z-2 does not involve significant differences in electrostatic potentials within different taxonomic groups

The low levels of protein variation observed in the course of evolution both within and between H2A.Z-1 and H2A.Z-2 variants are clearly emphasized when estimating the amino acid *p*-distances (*p*_AA_, Table 1). However, the amino acid sequence variation is slightly higher among H2A.Z-1 (0.058 ± 0.009 amino acid substitutions per site) compared to H2A.Z-2 or even to H2A.Z-e (0.032 ± 0.006 and 0.016 ± 0.007, respectively). Analysis of the variation at the nucleotide level (*p*_NT_, Table 1) reveals an inverse correlation with respect to the amino acid variation of H2A.Z-1, H2A.Z-2 and H2A.Z-e from early chordates (highest in this latter case with 0.148 ± 0.013 substitutions per site). The nature of the nucleotide variation is essentially synonymous and significantly greater than the nonsynonymous variation in all cases (**P < 0.001 in all codon-based Z-test comparisons). In an attempt to gain further insight into the evolutionary processes underlying H2A.Z-1 and H2A.Z-2 variability, we analyzed the amounts of total and synonymous nucleotide variation across both molecules as well as in the case of H2A.Z-e from early chordates. Such analyses provide useful information regarding the presence of functional constraints affecting the structural domains of proteins and have been successfully used in previous studies [26,28,32,39].

**Table 1:** Nucleotide and protein variation in the H2A variants analyzed.

	***p*_AA _(SE)**	***p*_NT _(SE)**	***p*_S _(SE)**	***p*_N _(SE)**	**Z-test^a^**	**R^b^**
H2A.Z-1	0.058 (0.009)	0.121 (0.008)	0.328 (0.016)	0.037 (0.006)	17.475**	1.3
H2A.Z-2	0.032 (0.006)	0.131 (0.011)	0.400 (0.020)	0.018 (0.003)	17.516**	1.4
H2A.Z-e	0.016 (0.007)	0.148 (0.013)	0.450 (0.031)	0.028 (0.009)	13.946**	1.2
Insects	0.024 (0.010)	0.203 (0.012)	0.611 (0.022)	0.037 (0.008)	24.826**	1.1
Fungi/Protists	0.309 (0.031)	0.385 (0.015)	0.710 (0.023)	0.260 (0.025)	12.886**	0.8
Overall	0.083 (0.011)	0.236 (0.019)	0.648 (0.024)	0.077 (0.009)	30.354**	0.9

Average numbers of amino acid (*p*_AA_), nucleotide (*p*_NT_), synonymous (*p*_S_), and nonsynonymous (*p*_N_) nucleotide differences per site and Z-test of selection.

Note.- SE, standard error; ** *P *< 0.001

^a ^H_0_: *p*_N _= *p*_S_; H_1_: *p*_N _<*p*_S_

^b ^Average transition/transversion ratio

An additional insight into the evolution of H2A.Z-1 and H2A.Z-2 can be obtained from their overall variation. The values shown in Table 2, indicate the presence of an extensive silent divergence between histones H2A.Z-1, H2A.Z-2 and H2A.Z-e with very similar magnitudes (ranging form 0.668 to 0.691 synonymous substitutions/site). However, protein distances reveal a different picture that is characterized by the close proximity between H2A.Z-2 and H2A.Z-e (0.028 substitutions/site), which is lower than the distance between H2A.Z-1 and H2A.Z-e (0.038 substitutions/site) and significantly lower than the overall distance between H2A.Z-1 and H2A.Z-2 variants from vertebrates (0.053 substitutions/site). These results support the presence of a mechanism of functional diversification and differentiation which most likely fits a birth-and-death model of evolution. This is based on the extensive synonymous nucleotide variation observed within and between variants as well as on the strong purifying selection constraining H2A.Z-1 and H2A.Z-2 at the protein level, which is similar to what is observed for all other histone families studied until now [28,32]. Contrary to other multigene families, the contribution of concerted evolution (gene conversion or interlocus recombination) to H2A.Z-1 and H2A.Z-2 long-term evolution can be disregarded considering the different chromosomal location of both forms in all organisms studied (see Additional file 2). In addition, both H2A.Z-1 and H2A.Z-2 seem to be more closely related to H2A.Z-e from early chordates than to one another, supporting the idea of a common origin for both variants in early deuterostomes following a subsequent process of differentiation later on in evolution. Indeed, the constraints operating on H2A.Z-1 at the nucleotide level during this differentiation process seem to be somewhat more relaxed compared with those operating on H2A.Z-2 and H2A.Z-e. Our results also reveal that H2A.Z-2 is as divergent from H2A.Z-1 as they both are from canonical H2A, a divergence that at the nucleotide level operates in all instances over the same regions of the protein (loop 1 and 2, as well as the docking domain). This diversification between H2A.Z-1/H2A.Z-2 and canonical H2A should be clearly distinguished from the relaxation in the constraints bewtween H2A.Z-1 and H2A.Z-2 to which we are going to subsequently refer in the text.

**Table 2:** Nucleotide and protein variation between the H2A variants analyzed and between different taxonomic groups.

	**H2A.Z-1**	**H2A.Z-2**	**H2A.Z-e**	**Insects**	**Fungi/Protists**
H2A.Z-1		0.683/0.037	0.691/0.042	0.771/0.063	0.771/0.181
H2A.Z-2	0.053/0.220		0.668/0.034	0.718/0.061	0.720/0.174
H2A.Z-e	0.038/0.224	0.028/0.213		0.656/0.052	0.730/0.165
Insects	0.037/0.263	0.030/0.247	0.005/0.222		0.718/0.174
Fungi/Protists	0.204/0.348	0.200/0.328	0.186/0.327	0.187/0.325	

Average numbers of amino acid and nucleotide differences per site (*p*_AA_/*p*_NT_, below diagonal) and average numbers of synonymous and nonsynonymous differences per site (*p*_S_/*p*_N_, above diagonal).

### The nature of the selective constraints operating on H2A.Z-1 and H2A.Z-2 histones

At the protein level, the evolutionary mechanisms leading to the differential identity of H2A.Z-1 and H2A.Z-2 seem to be operating beyond a process of purifying selection. Therefore, we decided to investigate the levels of codon usage bias in both genes in order to analyze the effect of selection at the nucleotide level. Differences in codon bias are common between different histone multigene families, more or less independently of the particular organisms studied [28,40,41]. Indeed, our results reveal significant differences between the H2A variants analyzed, with H2A.Z-2 (47.122 ± 2.456) exhibiting a significantly higher bias than H2A.Z-1 (55.106 ± 1.098; *t*-test = -6.799, *P *= 0.000). Such results have important implications for the evolutionary constraints affecting both histone types at the nucleotide level. Interestingly, no such significant difference in codon bias has ever been previously reported between histone variants belonging to the same histone multigene family, due to the presence of a strong purifying selection acting at the protein level as a major evolutionary force that leads to an extensive and homogeneous silent variation at the nucleotide level [28,29,32-34]. Also, the codon bias analyses are in agreement with the previous results at the amino acid level indicating also that H2A.Z-1 is less constrained than H2A.Z-2 at the nucleotide level. Furthermore, histone H2A.Z-e from early chordates displays an intermediate overall codon bias magnitude (49.666 ± 2.546) which is not significantly different from either H2A.Z-1 or from H2A.Z-2 (*P *> 0.05 in Duncan multiple range-test). These results suggest that the differences in the intensity of the selective constraints operating on H2A.Z-1 and H2A.Z-2 probably arose during the differentiation of both variants from a common ancestor, represented here by H2A.Z-e from early chordates.

Among other potential causes, differences in codon usage bias can be related to the preferential use of certain preferred triplets encoding overrepresented amino acids in the proteins, as occurs in highly basic proteins such as histones [28,42]. The most abundant residues in H2A.Z-1 and H2A.Z-2 proteins are represented by glycine and alanine (GC-rich amino acids) and lysine and isoleucine (GC-poor amino acids). The presence of selection for such biased amino acids in these classes was thus analyzed by studying the correlation between the frequency of GC-rich and GC-poor amino acids with the genomic GC content (see Additional files 6 and 7). We found that correlations between GC content and the frequency of GC-rich amino acids and GC-poor amino acids were not significant neither in H2A.Z-1 nor H2A.Z-2 (Fig. 3B and Fig. 3C). Similarly, the correlations between GC content and the most represented amino acids in each class (*P *> 0.05 in all Spearman rank correlations) were not significant either, as indicated in Table 3. These results indicate a departure from the neutral model of molecular evolution, which hypothesizes that GC-rich and GC-poor amino acids will be positively and negatively correlated with genomic GC content, respectively [43-46].

**Table 3:** Correlations between GC content and the frequency of GC-rich and GC-poor amino acids

**Histone H2A**	**Spearman rank correlation coefficient, r_S_**	***P*-value**
**H2A.Z-1**		
Genomic GC vs. GAPW (GC-rich)	-0.425	0.080
Genomic GC vs. FYMINK (GC-poor)	0.054	0.832
Genomic GC vs. Alanine	-0.323	0.191
Genomic GC vs. Lysine	-0.030	0.906
**H2A.Z-2**		
Genomic GC vs. GAPW (GC-rich)	0.024	0.906
Genomic GC vs. FYMINK (GC-poor)	0.293	0.143
Genomic GC vs. Alanine	-0.098	0.635
Genomic GC vs. Lysine	0.268	0.185

An additional method to gauge the significance of mutation bias and selection at the nucleotide level involves the comparison of changes at first codon positions (all nonsynonymous) with changes at fourfold positions (all synonymous) in the most frequent residues in H2A.Z-1 and H2A.Z-2. Under the neutral model the nucleotide frequencies at both positions should not be significantly different [47]. Codons for glycine and alanine (GC-rich) contain G at first codon positions, whereas codons for lysine and isoleucine (GC-poor) have A at first codon positions. Analysis of the mean G + A content at first codon positions in H2A.Z-1 (74.94 ± 0.67) and H2A.Z-2 (77.49 ± 0.84) showed that their values were significantly larger than the mean G + A content at fourfold degenerate positions in H2A.Z-1 (41.76 ± 4.73) and H2A.Z-2 (44.88 ± 2.86), (H2A.Z-1, *t*-test = 44.627, *P *= 0.000; H2A.Z-2, *t*-test = 37.901, *P *= 0.000). The values are significantly different in all species analyzed (see Additional files 6 and 7). While the neutral model of molecular evolution predicts that amino acid and nucleotide compositions are driven by the underlying GC content as a result of mutation bias, our results strongly suggest that selection has acted to maintain high levels of glycine, alanine, lysine and isoleucine in H2A.Z-1 and H2A.Z-2 variants, biasing their nucleotide composition. Few studies have shown that natural selection is more important than mutation bias in determining amino acid composition of proteins [28,42,47-49]. In this regard, our observations with H2A.Z stand in contrast to the neutral model.

### The progressive differentiation of H2A variants is mediated by stepwise mutations

The analyses presented in this work indicate that selective constraints governing H2A.Z-1 and H2A.Z-2 evolution go far beyond the protein level, as shown by the significant differences detected in codon usage bias and the presence of selection for highly biased amino acid composition that influences the nucleotide composition. However, the specific mechanisms responsible for the subtle differentiation between both forms and the functional meaning of this process remain obscure. In order to define possible functional selective targets in these proteins we decided to look at the codon usage of the amino acids of the triresidue that defines the identity of vertebrate H2A.Z-1 (Thr/Ser/Val) and H2A.Z-2 (Ala/Thr/Ala). The codons involved, which are indicated near each of the H2A sequences analyzed in the phylogeny shown in Fig. 2, show a high degree of conservation of codon in each histone form and within each taxonomic group.

**Figure 4:**
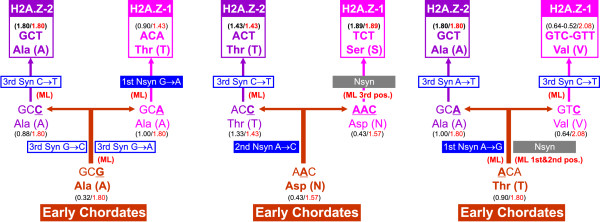
**Schematic representation of the stepwise mutation model leading from the early chordate triresidue to the mammalian H2A.Z-1 and H2A.Z-2 triresidues**. The model is presented independently for each of the positions in the triresidue. In the first step, the amino acid at a given position of the triresdue from early chordates is indicated. The second step involves the choice of either the H2A.Z-1 or the H2A.Z-2 pathway through a substitution of one of the positions in the codon which can result in a synonymous change or a residue replacement, configuring the triresidue in H2A.Z-1 and H2A.Z-2 proteins from non-mammalian vertebrates (usually *Xenopus *and zebrafish). The third step involves the transition of the already differentiated triresidues from non-mammalian vertebrates to those observed in mammalian H2A.Z-1 and H2A.Z-2 forms, through a single mutation in each of the three codons in most cases. The nature of the nucleotide substitutions mediating the transition from one step to another is indicated by open blue boxes (synonymous) and solid blue boxes (nonsynonymous) detailing the nucleotide change and the position at which it occurs. Multiple substitutions are indicated by solid grey boxes. The relative synonymous codon usage (RSCU) for each codon (black) is compared with the RSCU value for the preferred codon in that family (red) in each of the steps. In addition, substitutions fitting the Maximum composite likelihood estimation of the probability of substitution from one base to another simultaneously in H2A.Z-1 and H2A.Z-2 are identified in red as (ML).

Starting from early chordates, the amino acid triresidue defined as Ala/Asp/Thr eventually leads to the actual triresidues from both mammalian H2A.Z-1 and H2A.Z-2. Paying attention to the triplet encoding the first amino acid in the triresidue of H2A.Z-e from early chordates, we can trace a stepwise mutation pathway leading to the actual residue in the corresponding position of mammalian H2A.Z-1 and H2A.Z-2. For instance the first amino acid in the triresidue from early chordates is alanine (encoded by GCG), which undergoes two synonymous mutations leading to the corresponding actual residue in mammalian H2A.Z-2 (alanine, GCT), with an intermediate synonymous mutation step represented by H2A.Z-2 from amphibians (*Xenopus*, alanine GCC). Similarly, the pathway leading to the first residue of H2A.Z-1 would involve an intermediate synonymous mutation step in amphibians (*Xenopus*, alanine, GCA) and a final nonsynonymous step in the first codon position leading to mammalian H2A.Z-1 (threonine, ACA). It is important to point out that only one nucleotide at a given position in the codon changes in every step, with the remaining two positions being invariable. This mechanism is schematically shown in Fig. 4 which outlines the pathways starting from the amino acids in the triresidue from early chordates to the triresidue from mammalian H2A.Z-1 and H2A.Z-2, through an intermediate step represented by nonmammalian vertebrates (amphibians and zebrafish). This phenomenom reveals a very unique evolutionary process in which the evolution of the whole protein, leading either towards H2A.Z-1 or H2A.Z-2, is driven by the composition of the triresidue both at the amino acid and at the nucleotide levels.

Although the overall mutation pathway is stepwise, there are two instances in which more than one nucleotide change occurs in the same step: the change from AAC (Asp) to TCT (Ser) in the second amino acid of the triresidue, and from ACA (Thr) to GTC (Val) in the third amino acid of the triresidue. These multiple changes occur in the pathway towards H2A.Z-1, further illustrating the less constrained status of this variant compared to H2A.Z-2. Indeed, the pathways leading towards H2A.Z-2 involve an overall number of two amino acid replacements and four synonymous subtitutions, compared to the three amino acid replacements and two synonymous substitutions in the case of the H2A.Z-1. It is important to note here that the pathway leading towards H2A.Z-2 always ends using the triplet with higher relative synonymous codon usage within each of the three residues, while the codons in the case of H2A.Z-1 are always less preferred with the exception of serine. It is thus possible to identify a trend underlying the codon usage across these pathways, starting with triplets with low relative synonymous codon usage (RSCU) in the triresidue from early chordates, triplets with medium RSCU in the second step and triplets with maximum RSCU in the case of H2A.Z-2 and minimum RSCU in H2A.Z-1. Additional support for the higher levels of variation within H2A.Z-1 can be obtained from the estimation of the pattern of nucleotide substitutions shown in Table 4. By examining the overall number of nucleotide substitutions involved in the stepwise mutation model previously described, we found that from the six nucleotide changes involved in the pathway leading to mammalian H2A.Z-2, two reach maximum levels of probability as estimated in Table 4. In contrast, the same observation is made in six out of the nine nucleotide changes leading to H2A.Z-1. This suggests that this variant accumulates a sufficiently large number of nucleotide changes as to influence the maximum composite likelihood estimation of the probability of substitution of one nucleotide for another in H2A.Z-1 and H2A.Z-2.

**Table 4:** Maximum composite likelihood estimation of the probability of substitution in H2A.Z-1 and H2A.Z-2.

	**A**	**T**	**C**	**G**
A	-	4.45	5.03	8.82
T	5.40	-	22.16	5.52
C	5.40	19.61	-	5.52
G	8.62	4.45	5.03	-

Maximum composite likelihood estimation of the probability of substitution from one base (row) to another base (column) instantaneously.

## Conclusion

The function of histone H2A.Z in gene activation/silencing is still an important topic in chromatin research, as no clear mechanism for its structural and functional variability has yet been proposed. In this regard, the presence of two different H2A.Z forms is especially interesting [25]. Although very little is known about H2A.Z-2, the results presented in this work definitively reveal that its existence is not a whim of random genetic drift. The functional significance of H2A.Z-2 is still obscure, however our group has been able to demonstrate the coexistence of both H2A.Z-1 and H2A.Z-2 in chicken and human tissues, and that significant differences in their mRNA expression levels exist and in this regard, it is very likely that the key to the existence of these two functional H2A.Z forms resides within their promoter regions (manuscript in preparation). Our analyses demonstrate not only that H2A.Z-2 is subject to a strong purifying selection (as most histones are) but that in fact it is significantly more evolutionarily constrained than H2A.Z-1.

Nevertheless, it appears that this selection does not proceed in conventional ways. While phylogenetic and evolutionary analyses reveal a typical process of birth-and-death evolution with strong purifying selection leading to the differentiation of H2A family members [28], an almost identical primary structure has been conserved between H2A.Z-1 and H2A.Z-2 except for three amino acid differences. This is surprising considering that the two forms occupy different chromosomal locations (as revealed by the in silico analyses performed in the present work) and that they have resulted from a progressive differentiation across vertebrates starting from a common ancestor early in chordate evolution. The main evolutionary constraints directing the limited amino acid variation between H2A.Z-1 and H2A.Z-2 are primarily acting at the nucleotide level. This defines a process of stepwise mutation change in the codons constituting the triresidue which mirrors H2A.Z-1 and H2A.Z-2 evolution.

According to Clapier *et al*., the amino acid sequence changes observed in the protein variants throughout the highly constrained evolution of histones, are of little structural but decisive functional consequences [50]. Indeed, is worthwhile to mention that knocking out H2A.Z-1 in mice results in lethality and therefore (at least during early development), H2A.Z-2 cannot replace H2A.Z-1 (either in terms of abundance or function). In the instance of H2A.Z-1 and H2A.Z-2, the difference in amino acid sequence variability is minimal and it affects only three residues. Yet, we have observed a much closer proximity of the constraints imposed at the nucleotide level between the sequence of the genes encoding H2A.Z-2 and the histone H2A.Z ancestor (H2A.Z-e) when compared to H2AZ-1. This suggests that in the transition from chordates to vertebrates, the H2A.Z-1 has arisen to acquire a novel, or most likely complementary functions.

## Methods

### Mining of H2A nucleotide data

A total of 109 nonredundant H2A nucleotide coding sequences available from eukaryotes was collected from the histone database [51] and GenBank through BLAST searches, including 64 canonical H2A sequences, 30 H2A.X sequences, 8 macro H2A sequences, and 7 H2A.Bbd sequences (see Additional file 1). Additional data mining performed on complete and draft genome databases resulted in the identification of 69 nucleotide sequences encoding H2A.Z-1 and H2A.Z-2 histone variants from eukaryotes (see Additional file 2). In vertebrates, these variants were identified based on the differences shown in three residues (triresidue) characteristic either of H2A.Z-1 (21 sequences identified) or H2A.Z-2 (26 sequences identified), correcting sequence nomenclature when necessary (see Additional file 2). Given that sequences from early chordates contain mixed characteristics of both H2A.Z-1 and H2A.Z-2, these were called H2A.Z-e (4 sequences).

### Variation in H2A.Z-1 and H2A.Z-2 histone variants

Nucleotide coding sequences were aligned on the basis of their translated amino acid sequences using the BioEdit and CLUSTAL_X programs with the default parameters [52,53]. A bar chart representation was used in order show the frequency of each residue at every position of the alignment of vertebrate H2A.Z-1 and H2A.Z-2 forms using the LogoBar program [54]. The 3D structures of H2A.Z-1 and H2A.Z-2 proteins from vertebrates as well as H2A.Z-e from early chordates were modeled using the coordinates determined for the crystal structure of a nucleosome particle containing the variant histone H2A.Z-1 from human (PDB accession code 1F66) as a reference [19]. Evaluation of model qualities in homology modeling was performed by two approaches: 1) GROMOS empirical force energy to estimate the local quality of the predicted structure, with the y-axis representing the energy for each amino acid of the protein (negative and positive energy values represent favorable and unfavorable energy environments, respectively, for a given amino acid); 2) Verify3D to analyze the compatibility of an atomic model with its own amino acid sequence, in which the y-axis represents the average profile score for each residue in a 21-residue sliding window with scores ranging from -1 (bad score) to +1 (good score). All modeling and evaluation analyses were performed using the SWISS-MODEL workspace [55] and structures were rendered using the MacPyMOL program [56].

The comparisons between H2A protein variants with respect to their electrostatic properties were conducted in the webPIPSA pipeline [57], starting with a set of 3D structures modeled for all proteins listed in Additional file 2 and using human H2A.Z-1 as a reference. Electrostatic potentials were calculated using the University of Houston Brownian Dynamics (UHBD) program [58], and the absolute distances calculated from the similarity indices for the electrostatic potentials were represented in a colorized matrix and an epogram (tree representation of the relationships among potentials). The representation of the electrostatic potentials in the modeled 3D structures was implemented with the VMD program [59].

The extent of nucleotide and amino acid divergence between sequences was estimated using uncorrected differences (*p*-distance) as this distance is known to give better results than more complicated methods when the number of sequences is large and the number of positions used is relatively small, because of its smaller variance [60]. The numbers of synonymous and nonsynonymous nucleotide differences per site were computed by means of the modified Nei-Gojobori method [61]. Distances were estimated using the complete-deletion option in all cases and standard errors were calculated by the bootstrap method (1000 replicates).

All molecular and evolutionary analyses in this work were conducted using the program MEGA ver. 4.0 [62], as well as the calculation of amino acid and nucleotide frequencies, the relative synonymous codon usage (RSCU) and the maximum composite likelihood estimation of the nucleotide substitution patterns. The codon usage bias in H2A variants was referred to as the effective number of codons (ENC), which ranges from 61 (no bias) to 20 (maximum bias) and does not need any previous information on codon usage preferences in the genomes analyzed [63]. The analysis of the nucleotide variation across the different protein domains of H2A variants was performed by estimating the proportion (*p*) of nucleotide sites at which the two sequences being compared are different and the numbers of synonymous substitutions per site (*p*_*S*_), following a sliding-window approach (window length of 20 bp and step size of 5 bp for *p*, window length of 5 bp and step size of 1 bp for *p*_*S*_) implemented in the program DnaSP ver. 4.0 [64].

### Gauge of selection and selective constraints acting on H2A.Z-1 and H2A.Z-2 variants

The presence and nature of selection was studied following two strategies: 1) using the codon-based Z-test for selection [60] comparing the numbers of synonymous and nonsynonymous substitutions per site [61] in H2A genes, establishing the alternative hypothesis as H_1_: *p*_*N *_<*p*_*S *_and the null hypothesis as H_0_: *p*_*N *_= *p*_*S*_; 2) analyzing deviations from neutrality following two different approaches. First, the influence of selection on overrepresented amino acids was revealed by determining the correlation between the genomic GC content and the proportion of GC-rich (GAPW) and GC-poor amino acids (FYMINK). In this case, while GC-rich amino acids will be positively correlated with genomic GC content and vice-versa under the neutral model [43-46], they will not show any correlation if they are influenced by selection. In both cases, the GC content at fourfold degenerate positions was assumed to represent the genomic GC content, given that the latter has already been shown to be a good approximation of the former [65] and was also used as a good approximation to the neutral expectation. Correlations were computed using Spearman rank correlation analyses and statistical significance was assessed using standard regression analyses. Second, the effect of mutation and selection bias at the nucleotide level was studied by comparing nucleotide frequencies at first codon positions (always nonsynonymous in the case of the residues studied here) and at fourfold positions (always synonymous). Under the neutral model, nucleotide frequencies should not be significantly different between both positions [47].

### Inference of the phylogenetic relationships among H2A variants

Phylogenetic trees were reconstructed from the obtained *p*-distances using the neighbor-joining method [66]. To assess that our results are not dependent on this choice, phylogenetic inference analyses were completed by the reconstruction of maximum-parsimony trees [67] using the close-neighbor-interchange (CNI) search method. The bootstrap [68] and the interior branch-test [67,69] methods were combined in order to test the reliability of the trees, producing the bootstrap probability (BS) and the confidence probability (CP) values for each internal branch, assuming BP > 80% and CP ≥ 95% as statistically significant [70]. Histone H2A sequences of the diplomonad protist *Giardia *were used as outgroups, given that this lineage is believed to be the first to diverge from all other eukaryotes [71].

## Authors' contributions

JME-L conceived the study, and participated in its design and coordination and drafted the manuscript. RG-R participated in the phylogenetic studies of histone H2A. DD participated in the molecular evolutionary analysis and helped to draft the manuscript. TI carried out analysis on H2A.Z variation. JA conceived the study, and participated in its design and coordination and helped to draft the manuscript. All authors read and approved the final manuscript.

## Supplementary Material

Additional File 1 **GenBank Accession numbers for the histone H2A sequences used in the present work**. The data provided represent the accession numbers for H2A sequences used in this work, including canonical H2A genes and the variants H2A.Bbd, macro H2A, and H2A.X. The ANNOTATION field denotes: gene sequences newly isolated from draft genomes (In silico), and gene sequences predicted as H2A, H2A.Bbd and H2A.X from databases and draft/complete genomes data (PRED). Click here for file

Additional File 2 **GenBank Accession numbers for the histone variants H2A.Z-1, H2A.Z-2 and H2A.Z-e used in the present work**. The data provided include the accession numbers for the H2A.Z variants used in this work. The ANNOTATION field denotes: gene sequences newly isolated from draft genomes (In silico), gene sequences predicted as H2A, H2A.Z-1 and H2A.Z-2 from databases and draft/complete genomes data (PRED), sequences defined either as H2A.Z-1 or H2A.Z-2 by the present analyses (a), sequences defined as H2A by the present analyses (b) and sequences whose annotation either as H2A.Z-1 or H2A.Z-2 has been corrected by the present work (c). Click here for file

Additional File 3 **Phylogenetic neighbor-joining tree showing the phylogenetic relationships among histone H2A.Z-1 and H2A.Z-2 protein forms**. The reconstruction was carried out by calculating theee evolutionary amino acid *p*-distances from the H2A sequences of all the organisms analyzed (see Additional file 2). Histone H2A.Z-1 variants are indicated in pink, H2A.Z-2 variants are indicated in purple and H2A.Z-e sequences from early chordates are indicated in brown. Histone variants from protostomes, plants and fungi/protists are indicated in blue, while the root of the tree is labeled in black. Variant sequences predicted from databases and complete/draft genomes data are indicated by PRED near the species name. Numbers for interior nodes indicate BP/CP confidence values. Numbers in colored boxes and in boldface account for the bootstrap values obtained in the reconstruction of the maximum parsimony trees using all the informative positions in the alignment. Confidence values were based on 1000 replications and are only shown if at least one of the values is >50%. Click here for file

Additional File 4 **Graphical representation of the amino acid variation between H2A.Z-1 and H2A.Z-2 variants**. **A **Protein logos representation of the overall amino acid variation at each position of the alignment of H2A.Z-1 and H2A.Z-2 variants from vertebrates. The size of the bars is proportional to the frequency for a given amino acid and the overall height is proportional to the conservation of the sites. Colors were assigned to amino acids according to their physical and chemical structural characteristics (red, acidic; blue, basic; green, polar uncharged; purple, nonpolar hydrophobic). **B **Tertiary structures modeled for H2A.Z-1, H2A.Z-2 and H2A.Z-e from early chordates are shown below the protein logos representation, indicating the quality of the modeling process based on amino acid energies (gromos) and the compatibility of the 3D atomic models with the corresponding protein sequences (verify3d) at each amino acid position. Residues encompassing variation in the energy environment of the atomic model, including polymorphic positions between H2A.Z-1/H2A.Z-2 and H2A.Z-e from early chordates (indicated below in red) are highlighted in the 3D figures as well as in the nearby graphs. Click here for file

Additional File 5 **Electrostatic distances and potentials in different H2A.Z proteins**. **A **Electrostatic distances calculated from the similarity indices for the electrostatic potentials of histone H2A variants represented in a color coded matrix (heat map). The distance between similarity indices (*SI*) of two molecules (*a *and *b*) is defined as $D_{a,b}=\sqrt{2-2SI_{a,b}}$. The color code, as well as the number of comparisons for each distance interval are indicated in the key/histogram. The tree along the side of the image assembles the proteins into groups of similar electrostatic potentials (epogram), with discontinuous black lines delimiting three different groups of similarity with respect to human H2A.Z-1. **B **Representation of the electrostatic potentials for three representative H2A molecules belonging to different groups of similarity as defined in the epogram. Negatively charged surfaces are red and positively charged surfaces are blue, colors were assigned to amino acids according to their physical and chemical structural characteristics as in Supplementary Figure 2. The residue occupying the second position in the triresidue is indicated in each case. Click here for file

Additional File 6 **Genomic GC content, amino acid composition and A+G content at first codon positions and fourfold degenerate positions in the H2A.Z-1 genes analyzed**. The data provided was used for gauging the presence of selection acting on H2A.Z-1 genes. Click here for file

Additional File 7 **Genomic GC content, amino acid composition and A+G content at first codon positions and fourfold degenerate positions in the H2A.Z-2 genes analyzed**. The data provided was used for gauging the presence of selection acting on H2A.Z-2 genes. Click here for file
