# Supplementary material for: The evolutionary differentiation of two histone H2A.Z variants in chordates (H2A.Z-1 and H2A.Z-2) is mediated by a stepwise mutation process that affects three amino acid residues
Source: BMC Evol Biol. 2009 Feb 4;9:31. doi: 10.1186/1471-2148-9-31 (PMC2644675; doi:10.1186/1471-2148-9-31)
Supplement: Additional File 1 — GenBank Accession numbers for the histone H2A sequences used in the present work. The data provided represent the accession numbers for H2A sequences used in this work, including canonical H2A genes and the variants H2A.Bbd, macro H2A, and H2A.X. The ANNOTATION field denotes: gene sequences newly isolated from draft genomes (In silico), and gene sequences predicted as H2A, H2A.Bbd and H2A.X from databases and draft/complete genomes data (PRED). [file 1471-2148-9-31-S1.doc]

**Additional file 1: GenBank Accession numbers for the histone H2A sequences used in the present work including canonical H2A genes and the variants H2A.Bbd, macro H2A, and H2A.X.** The ANNOTATION field denotes: gene sequences newly isolated from draft genomes (In silico), and gene sequences predicted as H2A, H2A.Bbd and H2A.X from databases and draft/complete genomes data (PRED).

| **TAXONOMIC GROUP** | **SPECIES** | **GENE** | **ACCESSION NUMBER (nucleotide)** | **ANNOTATION** |
| --- | --- | --- | --- | --- |
| **ANIMALS** |  |  |  |  |
| TRIPLOBLASTS (bilaterians) |  |  |  |  |
| **Deuterostomes** |  |  |  |  |
| Birds |  |  |  |  |
|  | Cairina moschata (Muscovy Duck) | H2A | X14730 |  |
|  | Gallus gallus (Chicken) | H2A (1) | X02218 |  |
|  |  | H2A (2) | U38931 |  |
|  |  | macroH2A1.1 | AF058445 |  |
|  |  | macroH2A1.2 | AF058446 |  |
| Mammals |  |  |  |  |
|  | Bos taurus (Cattle) | H2A.Bbd | NM_001075905 |  |
|  | Felis catus (Cat) | H2A.Bbd | AANG01525054 | In silico |
|  | Homo sapiens (Human) | H2A (1) | X57138 |  |
|  |  | H2A (2) | AC004854 |  |
|  |  | H2A (3) | NM_003509 |  |
|  |  | H2A (4) | NM_003510 |  |
|  |  | H2A (5) | NM_003511 |  |
|  |  | H2A (6) | NM_003514 |  |
|  |  | H2A (7) | NM_021064 |  |
|  |  | H2A (8) | BC001193 |  |
|  |  | H2A.X | BC004915 |  |
|  |  | H2A.Bbd | AF254576 |  |
|  |  | macroH2A1.1 | AF044286 |  |
|  |  | macroH2A1.2 | AF041483 |  |
|  |  | macroH2A2.2 | NP_061119 |  |
|  | Macaca mulatta (Rhesus Monkey) | H2A.Bbd | XM_001091351 | Pred (Bbd) |
|  | Mus musculus (Mouse) | H2A (1) | M33988 |  |
|  |  | H2A (2) | X16148 |  |
|  |  | H2A.X | BC005468 |  |
|  |  | H2A.Bbd | XM_988138 | Pred (Bbd) |
|  |  | macroH2A1.2 | AF171080 |  |
|  |  | macroH2A2.2 | AF336305 |  |
|  | Pan troglodytes (Chimpanzee) | H2A.Bbd | XM_001145032 | Pred (Bbd) |
|  |  | H2A.X | XM_522264 | Pred (X) |
|  | Rattus norvegicus (Rat) | H2A | U95113 |  |
|  |  | H2A.Bbd | XM_229093 | Pred (Bbd) |
|  |  | macroH2A1.2 | U79139 |  |
| Fishes |  |  |  |  |
|  | Carassius auratus gibelio (Carp) | H2A | AF315728 |  |
|  |  | H2A.X | AF315729 | Pred (X) |
|  | Danio rerio (Zebrafish) | H2A.X | BC046078 |  |
|  | Oncorhynchus mykiss (Rainbow Trout) | H2A | X01064 |  |
| Amphibians |  |  |  |  |
|  | Bufo gargarizans (Asian Toad) | H2A (1) | AF255739 |  |
|  |  | H2A (2) | U70133 |  |
|  |  | H2A (3) | AF255740 |  |
|  | Xenopus laevis | H2A | X03018 |  |
|  |  | H2A.X | BC056660 | Pred (X) |
| Echinoderms |  |  |  |  |
|  | Paracentrotus lividus | H2A | M25281 |  |
|  |  | H2A | Y09062 |  |
|  | Psammechinus miliaris | H2A2.1 | M14141 |  |
|  |  | H2A2.2 | M14140 |  |
|  | Strongylocentrotus purpuratus | H2A-late (1) | V01357 |  |
|  |  | H2A-late (2) | X06642 |  |
| Protostomes |  |  |  |  |
| Insects |  |  |  |  |
|  | Anopheles gambiae | H2A.X | AAAB01008986 | In silico |
|  | Bombyx mori (Silkworm) | H2A | AB001052 |  |
|  | Chironomus thummi | H2A (1) | X56335 |  |
|  |  | H2A (2) | X72803 |  |
|  | Drosophila hydei | H2A | X52576 |  |
|  | Drosophila melanogaster | H2A | AE014134 | Pred |
|  | Rhynchosciara americana | H2A | AF378198 |  |
| Crustaceans |  |  |  |  |
|  | Asellus aquaticus | H2A | AJ238321 |  |
|  | Tigriopus californicus | H2A | M84797 |  |
|  |  |  |  |  |
|  |  |  |  |  |
| Molluscs |  |  |  |  |
|  | Mytilus californianus (California Mussel) | H2A | AY267759 |  |
|  | Mytilus chilensis (Chilean Mussel) | H2A | AY267756 |  |
|  | Mytilus edulis (Blue Mussel) | H2A | AY267757 |  |
|  | Mytilus galloprovincialis (Mediterranean Mussel) | H2A | AY267755 |  |
|  | Mytilus trossulus (Common Blue Mussel) | H2A | AY267758 |  |
| Annelids/Echiurids |  |  |  |  |
|  | Chaetopterus variopedatus | H2A | AF007904 |  |
|  | Platynereis dumerilii | H2A | X53330 |  |
|  | Urechis caupo | H2A | X58895 |  |
| DIPLOBLASTS |  |  |  |  |
| Cnidarians |  |  |  |  |
|  | Acropora formosa (Coral) |  | L11067 |  |
| PLANTS |  |  |  |  |
|  | Arabidopsis thaliana | H2A (1) | AF204967 |  |
|  |  | H2A (2) | AF204968 |  |
|  |  | H2A.X | BT002503 |  |
|  | Brassica napus (Rape) | H2A | L11067 |  |
|  | Cicer arietinum (Chickpea) | H2A.X | AJ006768 | Pred (X) |
|  | Euphorbia esula | H2A | AF242311 |  |
|  | Lilium longiflorum | H2A (1) | AB003781 |  |
|  |  | H2A (2) | AB003782 |  |
|  | Petroselinum crispum (Parsley) | H2A | X53831 |  |
|  | Picea abies | H2A.X | X67819 |  |
|  | Pinus taeda | H2A | AF013803 |  |
|  | Pisum sativum (Pea) | H2A | U10041 |  |
|  | Triticum aestivum (Wheat) | H2A | D38087 |  |
|  | Zea mays (Maize) | H2A | U08225 |  |
| FUNGI |  |  |  |  |
|  | Agaricus bisporus | H2A.X | AJ293758 | Pred (X) |
|  | Ashbya gossypii | H2A.X | NM_210212 | Pred (X) |
|  | Aspergillus niger | H2A.X | Y15320 | Pred (X) |
|  | Botrytis fuckeliana | H2A.X | AJ006959 | Pred (X) |
|  | Candida albicans | H2A.X | AACQ01000147 | In silico |
|  | Candida glabrata | H2A.X | XM_448713 | Pred (X) |
|  | Debaryomyces hansenii | H2A.X | XM_461153 | Pred (X) |
|  | Emericella nidulans | H2A.X | M18258 | Pred (X) |
|  | Kluyveromyces lactis | H2A.X | XM_455680 | Pred (X) |
|  | Neurospora crassa | H2A.X | AABX01000389 | In silico |
|  | Saccharomyces cerevisiae (Yeast) | H2A.X (1) | Z26494 | Pred (X) |
|  |  | H2A.X (2) | V01304 | Pred (X) |
|  | Schizosaccharomyces pombe (Fission Yeast) | H2A.X | X05221 | Pred (X) |
|  |  | H2A.X | X05220 | Pred (X) |
|  |  | H2A.X | Z97209 | Pred (X) |
|  |  | H2A.X | M11494 | Pred (X) |
|  | Yarrowia lipolytica | H2A.X | XM_504426 | Pred (X) |
| PROTISTS |  |  |  |  |
|  | Chlamydomonas reinhardtii | H2A | L41841 |  |
|  | Crithidia fasciculata | H2A | U87597 |  |
|  | Cryptosporidium parvum | H2A.X | AAEE01000003 | In silico |
|  | Euglena gracilis | H2A | X73147 |  |
|  | Giardia intestinalis | H2A | AF139873 |  |
|  | Giardia lamblia | H2A | AACB01000030 | Pred |
|  | Leishmania donovani | H2A | X60054 |  |
|  | Plasmodium falciparum | H2A | M86865 |  |
|  | Toxoplasma gondii | H2A.X | AY631392 | Pred (X) |
|  | Tetrahymena thermophila | H2A.X | L18892 |  |
|  | Trypanosoma cruzi | H2A | X83272 |  |
|  | Trypanosoma rangeli | H2A | AF169130 |  |
|  | Volvox carteri | H2A (1) | M31921 |  |
|  |  | H2A (2) | M31922 |  |
