# Supplementary material for: The evolutionary differentiation of two histone H2A.Z variants in chordates (H2A.Z-1 and H2A.Z-2) is mediated by a stepwise mutation process that affects three amino acid residues
Source: BMC Evol Biol. 2009 Feb 4;9:31. doi: 10.1186/1471-2148-9-31 (PMC2644675; doi:10.1186/1471-2148-9-31)
Supplement: Additional File 2 — GenBank Accession numbers for the histone variants H2A.Z-1, H2A.Z-2 and H2A.Z-e used in the present work. The data provided include the accession numbers for the H2A.Z variants used in this work. The ANNOTATION field denotes: gene sequences newly isolated from draft genomes (In silico), gene sequences predicted as H2A, H2A.Z-1 and H2A.Z-2 from databases and draft/complete genomes data (PRED), sequences defined either as H2A.Z-1 or H2A.Z-2 by the present analyses (a), sequences defined as H2A by the present analyses (b) and sequences whose annotation either as H2A.Z-1 or H2A.Z-2 has been corrected by the present work (c). [file 1471-2148-9-31-S2.doc]

**Additional file 2: GenBank Accession numbers for the histone variants H2A.Z-1, H2A.Z-2 and H2A.Z-e used in the present work.** The ANNOTATION field denotes: gene sequences newly isolated from draft genomes (In silico), **gene** sequences predicted as H2A, H2A.Z-1 and H2A.Z-2 from databases and draft/complete genomes data (PRED), sequences defined either as H2A.Z-1 or H2A.Z-2 by the present analyses (a), sequences defined as H2A by the present analyses (b) and sequences whose annotation either as H2A.Z-1 or H2A.Z-2 has been corrected by the present work (c).

| **TAXONOMIC GROUP** | **SPECIES** | **GENE** | **ACCESSION NUMBER (nucleotide)** | **ANNOTATION** |
| --- | --- | --- | --- | --- |
| **ANIMALS** |  |  |  |  |
| TRIPLOBLASTS (bilaterians) |  |  |  |  |
| **Deuterostomes** |  |  |  |  |
| Birds |  |  |  |  |
|  | Gallus gallus (Chicken) | H2A.Z-2 | V00414 | In silico (a,b) |
|  |  | H2A.Z-1 | 4/NM_001031374 | (c) |
| Mammals |  |  |  |  |
|  | Bos taurus (Cattle) | H2A.Z-1 | 4/NM_174809 |  |
|  |  | H2A.Z-2 | 6/NM_001038197 |  |
|  | Canis familiaris (Dog) | H2A.Z-2 | 16/XM_532724 | Pred |
|  |  | H2A.Z-1 (1) | 2/XM_535390 | Pred |
|  |  | H2A.Z-1 (2) | 32/XM_535671 | Pred (a) |
|  |  | H2A.Z-1 (3) | 32/XM_857355 | Pred (a) |
|  |  | H2A.Z-1 (4) | 32/XM_857381 | Pred (a) |
|  | Equus caballus (Horse) | H2A.Z-2 | 4/XM_001495899 | Pred (a,b) |
|  | Homo sapiens (Human) | H2A.Z-2 (1) | 7/NM_012412 |  |
|  |  | H2A.Z-2 (2) | 7/NM_138635 |  |
|  |  | H2A.Z-2 (3) | 7/NM_201436 |  |
|  |  | H2A.Z-1 | 4/NM_002106 |  |
|  | Macaca mulatta (Rhesus Monkey) | H2A.Z-2 | 3/XM_001093992 | In silico (a,b) |
|  |  | H2A.Z-1 (1) | 5/XM_001108067 | Pred |
|  |  | H2A.Z-1 (2) | 5/XM_001108128 | Pred |
|  |  | H2A.Z-1 (3) | 9/XP_001097247 | Pred (c) |
|  | Monodelphis domestica | H2A.Z-2 | 1/XM_001379779 | Pred (a,b) |
|  |  | H2A.Z-1 | 5/XM_001364009 | Pred (a,b) |
|  | Mus musculus (Mouse) | H2A.Z-2 (1) | 11/XM_907680 | Pred |
|  |  | H2A.Z-2 (2) | 11/XM_00147068 | Pred |
|  |  | H2A.Z-1 | 3/NM_016750 |  |
|  | Ovis aries (Sheep) | H2A.Z-2 | EE873683 | In silico (a,b) |
|  |  | H2A.Z-1 | NM_001009270 |  |
|  | Pan troglodytes (Chimpanzee) | H2A.Z-2 | 15/NW_001225258 | In silico (a,b) |
|  |  | H2A.Z-1 (1) | 8/XM_001163743 | Pred (a,b) |
|  |  | H2A.Z-1 (2) | 8/XM_519801 | Pred (a,b) |
|  | Rattus norvegicus (Rat) | H2A.Z-2 (1) | 14/NM_001106019 |  |
|  |  | H2A.Z-2 (2) | 4/XM_001056732 | Pred |
|  |  | H2A.Z-2 (3) | 18/XM_001058159 | Pred |
|  |  | H2A.Z-2 (4) | 4/XM_001062139 | Pred |
|  |  | H2A.Z-2 (5) | 6/XM_001070681 | Pred |
|  |  | H2A.Z-2 (6) | 6/XM_001080182 | Pred |
|  |  | H2A.Z-2 (7) | 18/XM_574181 | Pred |
|  |  | H2A.Z-1 | 2/NM_022674 |  |
|  | Sus scrofa (Pig) | H2A.Z-2 | EW362526 | In silico (a,b) |
|  |  | H2A.Z-1 | 8/NM_001123122 |  |
| Fishes |  |  |  |  |
|  | Danio rerio (Zebrafish) | H2A.Z-2 (1) | 5/NM_153644 |  |
|  |  | H2A.Z-2 (2) | 8/XM_702461 | Pred (a,b) |
|  |  | H2A.Z-1 | 10/NM_001043323 |  |
| Amphibians |  |  |  |  |
|  | Xenopus laevis | H2A.Z-2 (1) | NM_001092643 | (a,b) |
|  |  | H2A.Z-2 (2) | NM_001093882 | (a,b) |
|  |  | H2A.Z-1 (1) | NM_001088140 |  |
|  |  | H2A.Z-1 (2) | NM_001086059 |  |
|  | Xenopus tropicalis | H2A.Z-2 | NM_001016178 |  |
|  |  | H2A.Z-1 | NP_001005097 |  |
| Echinoderms |  |  |  |  |
|  | Strongylocentrotus purpuratus (Sea Urchin) | H2A.Z-e (1) | XM_786337 |  |
|  |  | H2A.Z-e (2) | XP_001176112 |  |
| Cephalochordates |  |  |  |  |
|  | Branchiostoma floridae (Amphioxus) | H2A.Z-e | Brafl1|72478|fgenesh2_pg.scaffold_38000135 | In silico (a,b) |
| Protostomes |  |  |  |  |
| Insects |  |  |  |  |
|  | Aedes aegypti | H2A.Z | XM_001658460 | (a) |
|  | Anopheles gambiae | H2A.Z | X/XM_310818 | (b) |
|  | Apis mellifera (Honey Bee) | H2A.V | LG7/XM_624164 | Pred |
|  | Bombyx mori (Silkworm) | H2A.V | NM_001047064 |  |
|  | Culex quinquefasciatus | H2A.Z | XM_001850088 | (a) |
|  | Drosophila melanogaster | His2Av | 3R/NM_079795 |  |
|  | Drosophila pseudoobscura | His2Av | 2/XM_001357925 | (2) |
|  | Nasonia vitripennis (Wasp) | H2A.Z | XM_001605015 | Pred |
|  | Tribolium castaneum | H2A.Z | LG2/XM_970375 | Pred (a,b) |
| Nematodes |  |  |  |  |
|  | Caenorhabditis briggsae | Htz1 | IV/XM_001671182 | (b) |
|  | Caenorhabditis elegans | Htz1 | IV/NM_068168 |  |
| DIPLOBLASTS |  |  |  |  |
| Cnidarians |  |  |  |  |
|  | Nematostella vectensis (sea anemone) | H2A.Z-e | XM_001638302 | Pred (a,b) |
| PLANTS |  |  |  |  |
|  | Arabidopsis thaliana | H2A.Z | 3/NM_115313 | (b) |
|  | Oryza sativa | H2A.Z | 3/NM_001057767) | (a,b) |
| FUNGI |  |  |  |  |
|  | Neurospora crassa | Htz1 | 5/XM_743054 |  |
|  | Saccharomyces cerevisiae (Yeast) | Htz1 | XV/AY558000 | (a,b) |
|  | Schizosaccharomyces pombe (Fission Yeast) | Htz1 | II/NM_001021524 | (a) |
| PROTISTS |  |  |  |  |
|  | Chlamydomonas reinhardtii | H2A.Z | XM_001693648 | (a) |
|  | Giardia intestinalis | H2A (root) | AF139873 |  |
|  | Tetrahymena thermophila | hv1 | X15548 |  |
