# Supplementary figures and images for: The evolutionary differentiation of two histone H2A.Z variants in chordates (H2A.Z-1 and H2A.Z-2) is mediated by a stepwise mutation process that affects three amino acid residues
Source: BMC Evol Biol. 2009 Feb 4;9:31. doi: 10.1186/1471-2148-9-31 (PMC2644675; doi:10.1186/1471-2148-9-31)

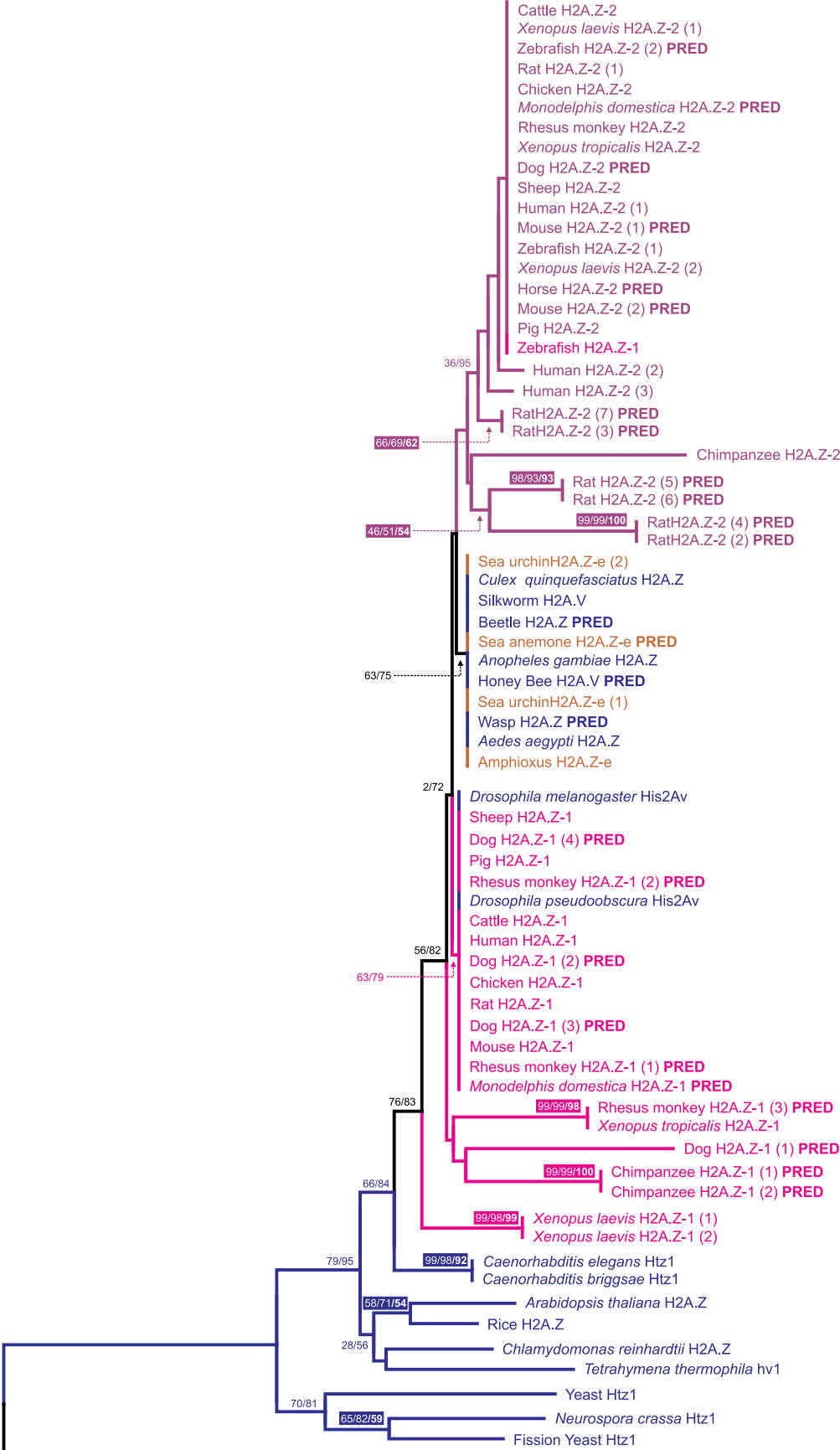

0.05

Supplement: Additional File 3 — Phylogenetic neighbor-joining tree showing the phylogenetic relationships among histone H2A.Z-1 and H2A.Z-2 protein forms. The reconstruction was carried out by calculating theee evolutionary amino acid p-distances from the H2A sequences of all the organisms analyzed (see Additional file 2). Histone H2A.Z-1 variants are indicated in pink, H2A.Z-2 variants are indicated in purple and H2A.Z-e sequences from early chordates are indicated in brown. Histone variants from protostomes, plants and fungi/protists are indicated in blue, while the root of the tree is labeled in black. Variant sequences predicted from databases and complete/draft genomes data are indicated by PRED near the species name. Numbers for interior nodes indicate BP/CP confidence values. Numbers in colored boxes and in boldface account for the bootstrap values obtained in the reconstruction of the maximum parsimony trees using all the informative positions in the alignment. Confidence values were based on 1000 replications and are only shown if at least one of the values is >50%. [file 1471-2148-9-31-S3.pdf]

**A**

bits

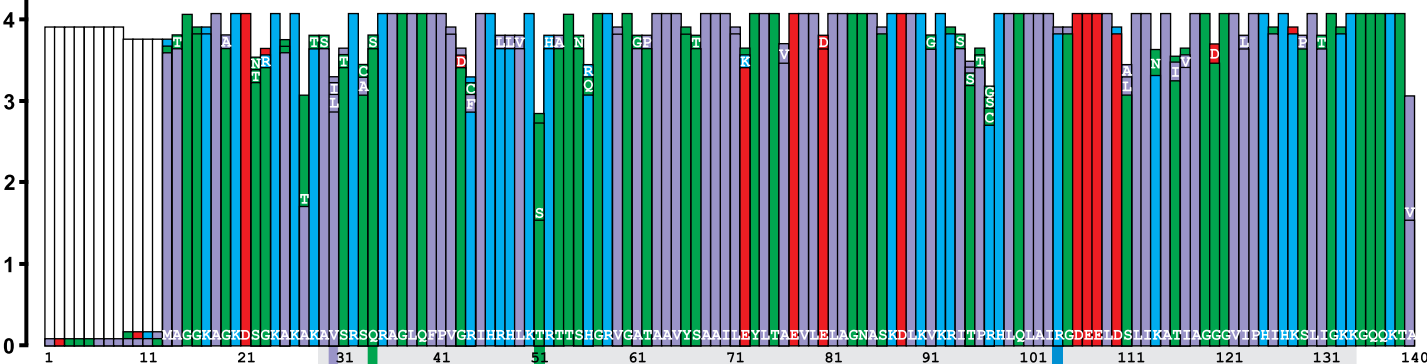**B**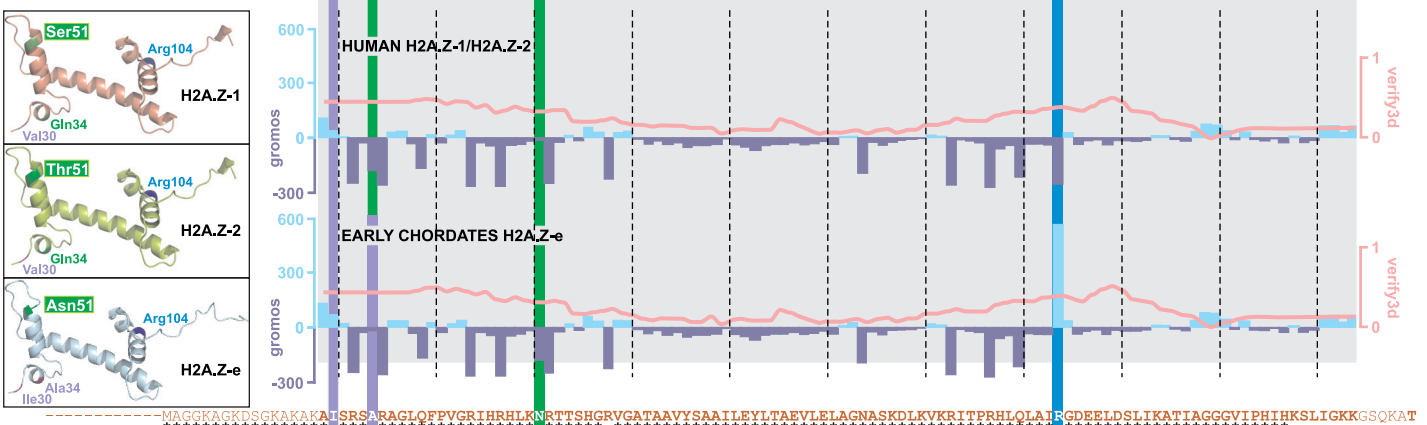

Supplement: Additional File 4 — Graphical representation of the amino acid variation between H2A.Z-1 and H2A.Z-2 variants. A Protein logos representation of the overall amino acid variation at each position of the alignment of H2A.Z-1 and H2A.Z-2 variants from vertebrates. The size of the bars is proportional to the frequency for a given amino acid and the overall height is proportional to the conservation of the sites. Colors were assigned to amino acids according to their physical and chemical structural characteristics (red, acidic; blue, basic; green, polar uncharged; purple, nonpolar hydrophobic). B Tertiary structures modeled for H2A.Z-1, H2A.Z-2 and H2A.Z-e from early chordates are shown below the protein logos representation, indicating the quality of the modeling process based on amino acid energies (gromos) and the compatibility of the 3D atomic models with the corresponding protein sequences (verify3d) at each amino acid position. Residues encompassing variation in the energy environment of the atomic model, including polymorphic positions between H2A.Z-1/H2A.Z-2 and H2A.Z-e from early chordates (indicated below in red) are highlighted in the 3D figures as well as in the nearby graphs. [file 1471-2148-9-31-S4.pdf]

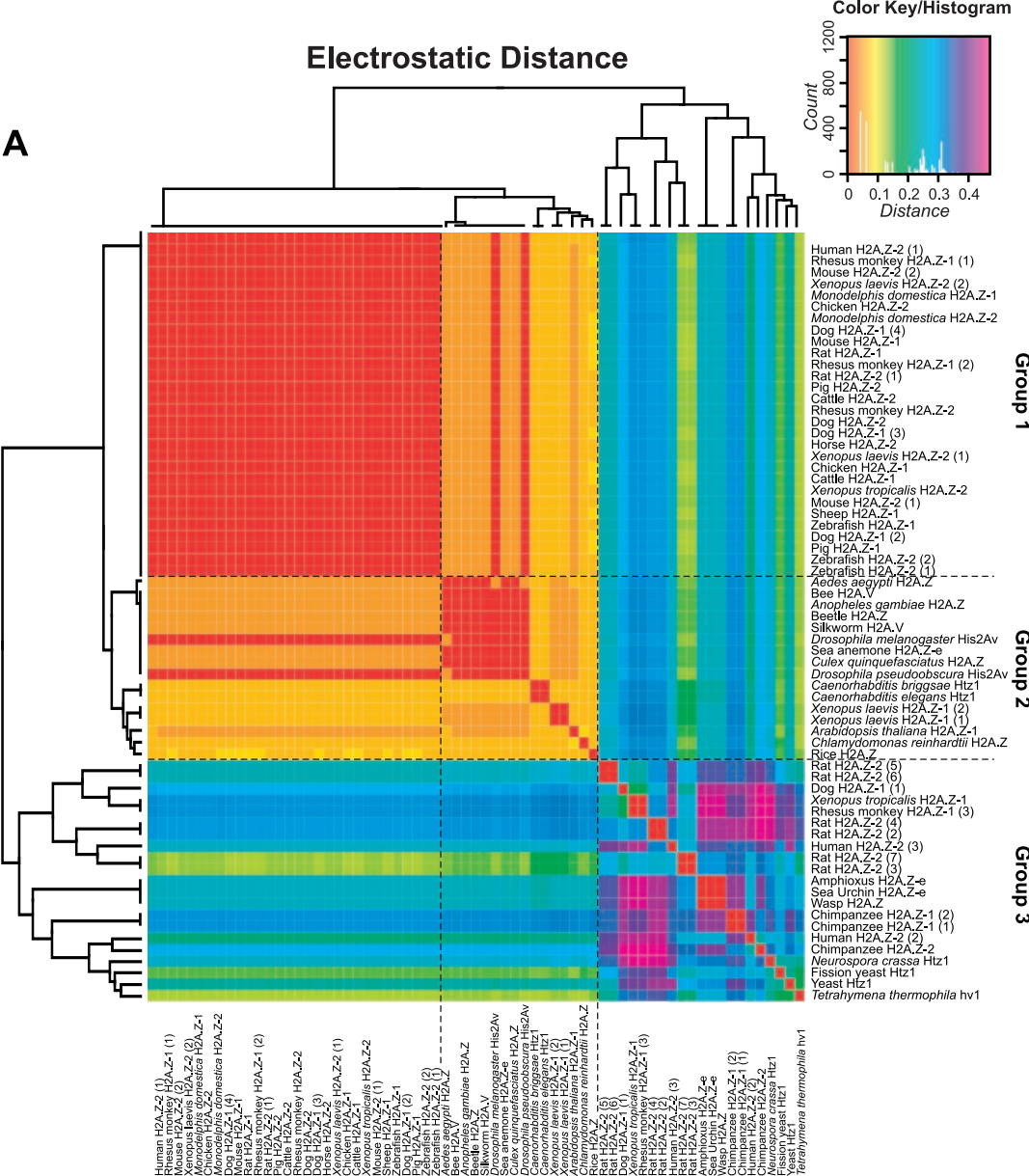

**B**

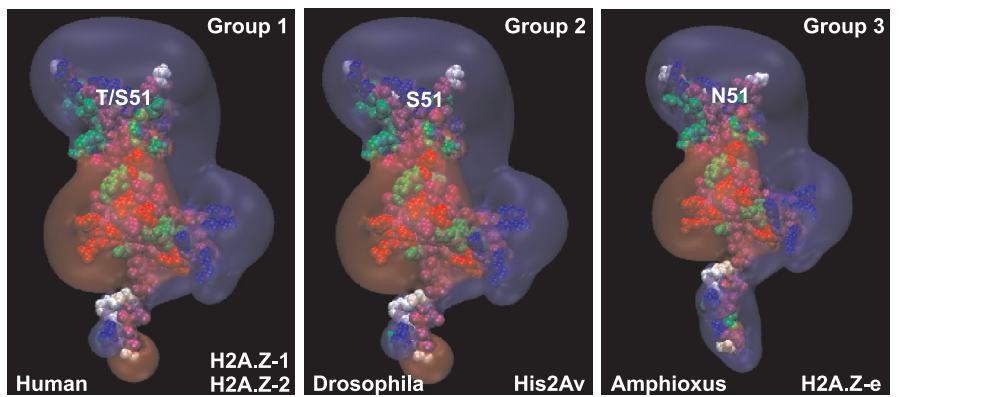

Supplement: Additional File 5 — Electrostatic distances and potentials in different H2A.Z proteins. A Electrostatic distances calculated from the similarity indices for the electrostatic potentials of histone H2A variants represented in a color coded matrix (heat map). The distance between similarity indices (SI) of two molecules (a and b) is defined as Da,b=2−2SIa,b. The color code, as well as the number of comparisons for each distance interval are indicated in the key/histogram. The tree along the side of the image assembles the proteins into groups of similar electrostatic potentials (epogram), with discontinuous black lines delimiting three different groups of similarity with respect to human H2A.Z-1. B Representation of the electrostatic potentials for three representative H2A molecules belonging to different groups of similarity as defined in the epogram. Negatively charged surfaces are red and positively charged surfaces are blue, colors were assigned to amino acids according to their physical and chemical structural characteristics as in Supplementary Figure 2. The residue occupying the second position in the triresidue is indicated in each case. [file 1471-2148-9-31-S5.pdf]
