# Supplementary material for: The evolutionary differentiation of two histone H2A.Z variants in chordates (H2A.Z-1 and H2A.Z-2) is mediated by a stepwise mutation process that affects three amino acid residues
Source: BMC Evol Biol. 2009 Feb 4;9:31. doi: 10.1186/1471-2148-9-31 (PMC2644675; doi:10.1186/1471-2148-9-31)
Supplement: Additional File 6 — Genomic GC content, amino acid composition and A+G content at first codon positions and fourfold degenerate positions in the H2A.Z-1 genes analyzed. The data provided was used for gauging the presence of selection acting on H2A.Z-1 genes. [file 1471-2148-9-31-S6.doc]

**Additional file 6: Genomic GC content, amino acid composition and A+G content at first codon positions and fourfold degenerate positions in the H2A.Z-1 genes analyzed*.**

|  | **fourfold G+C** | **GAPW** | **FYMINK** | **Gly (GGN)** | **Ala (GCN)** | **Lys (AAR)** | **Ile (ATD)** | **1^st^ pos. A+G** | **fourfold A+G** |
| --- | --- | --- | --- | --- | --- | --- | --- | --- | --- |
| Chicken H2A.Z-1 | 26.40 | 25.78 | 24.22 | 11.72 | 11.72 | 10.94 | 7.03 | 75.70 | 44.60 |
| Human H2A.Z-1 | 41.50 | 26.56 | 24.22 | 11.72 | 12.50 | 10.94 | 7.03 | 74.70 | 44.70 |
| Rhesus Monkey H2A.Z-1 (1) | 41.50 | 26.56 | 24.22 | 11.72 | 12.50 | 10.94 | 7.03 | 74.70 | 44.70 |
| Rhesus Monkey H2A.Z-1 (2) | 41.50 | 26.56 | 24.22 | 11.72 | 12.50 | 10.94 | 7.03 | 74.70 | 44.70 |
| Rhesus Monkey H2A.Z-1 (3) | 45.30 | 25.78 | 26.56 | 10.94 | 12.50 | 10.94 | 7.81 | 74.70 | 41.10 |
| Rhesus Monkey H2A.Z-1 (4) | 43.40 | 25.78 | 23.44 | 12.50 | 11.72 | 11.72 | 5.47 | 74.70 | 41.10 |
| Chimpanzee H2A.Z-1 (1) | 37.80 | 25.78 | 23.44 | 10.16 | 13.28 | 10.16 | 7.03 | 76.50 | 42.90 |
| Chimpanzee H2A.Z-1 (2) | 43.40 | 25.78 | 23.44 | 12.50 | 11.72 | 11.72 | 5.47 | 76.50 | 42.90 |
| Pig H2A.Z-1 | 43.40 | 26.56 | 24.22 | 11.72 | 12.50 | 10.94 | 7.03 | 74.70 | 42.90 |
| Cattle H2A.Z-1 | 41.50 | 26.56 | 24.22 | 11.72 | 12.50 | 10.94 | 7.03 | 74.70 | 44.60 |
| Sheep H2A.Z-1 | 39.60 | 26.56 | 24.22 | 11.72 | 12.50 | 10.94 | 7.03 | 74.70 | 44.70 |
| Dog H2A.Z-1 (1) | 45.20 | 26.56 | 24.22 | 11.72 | 12.50 | 10.94 | 7.03 | 74.70 | 46.40 |
| Dog H2A.Z-1 (2) | 45.20 | 26.56 | 24.22 | 11.72 | 12.50 | 10.94 | 7.03 | 74.70 | 46.40 |
| Dog H2A.Z-1 (3) | 45.20 | 26.56 | 24.22 | 11.72 | 12.50 | 10.94 | 7.03 | 74.70 | 46.40 |
| Dog H2A.Z-1 (4) | 43.40 | 26.56 | 24.22 | 11.72 | 12.50 | 10.94 | 7.03 | 73.80 | 44.60 |
| Mouse H2A.Z-1 | 43.40 | 26.56 | 24.22 | 11.72 | 12.50 | 10.94 | 7.03 | 74.70 | 46.40 |
| Rat H2A.Z-1 | 37.80 | 26.56 | 24.22 | 11.72 | 12.50 | 10.94 | 7.03 | 74.70 | 51.80 |
| M. domestica H2A.Z-1 | 22.20 | 28.03 | 25.03 | 11.36 | 13.64 | 11.36 | 7.58 | 75.60 | 50.80 |
| X. tropicalis H2A.Z-1 | 45.30 | 25.78 | 26.56 | 10.94 | 12.50 | 10.94 | 7.81 | 74.70 | 41.10 |
| **Average** | **40.68±6.27** | **26.39±0.54** | **24.39±0.85** | **11.62±0.52** | **12.48±0.45** | **11.00±0.33** | **6.98±0.59** | **74.94±0.67** | **44.88±2.86** |

* See Additional file 2 for GenBank accession numbers.
