# Supplementary material for: The evolutionary differentiation of two histone H2A.Z variants in chordates (H2A.Z-1 and H2A.Z-2) is mediated by a stepwise mutation process that affects three amino acid residues
Source: BMC Evol Biol. 2009 Feb 4;9:31. doi: 10.1186/1471-2148-9-31 (PMC2644675; doi:10.1186/1471-2148-9-31)
Supplement: Additional File 7 — Genomic GC content, amino acid composition and A+G content at first codon positions and fourfold degenerate positions in the H2A.Z-2 genes analyzed. The data provided was used for gauging the presence of selection acting on H2A.Z-2 genes. [file 1471-2148-9-31-S7.doc]

**Additional file 7: Genomic GC content, amino acid composition and A+G content at first codon positions and fourfold degenerate positions in the H2A.Z-2 genes analyzed*.**

|  | **fourfold G+C** | **GAPW** | **FYMINK** | **Gly (GGN)** | **Ala (GCN)** | **Lys (AAR)** | **Ile (ATD)** | **1^st^ pos. A+G** | **fourfold A+G** |
| --- | --- | --- | --- | --- | --- | --- | --- | --- | --- |
| Chicken H2A.Z-2 | 79.70 | 28.12 | 24.22 | 11.72 | 14.06 | 10.94 | 7.03 | 77.90 | 38.40 |
| Human H2A.Z-2 (1) | 30.50 | 28.12 | 24.22 | 11.72 | 14.06 | 10.94 | 7.03 | 77.90 | 38.30 |
| Human H2A.Z-2 (2) | 30.80 | 27.19 | 21.93 | 10.53 | 14.91 | 9.65 | 5.26 | 78.10 | 35.90 |
| Human H2A.Z-2 (3) | 31.90 | 24.50 | 24.30 | 9.80 | 11.76 | 8.82 | 8.82 | 78.10 | 38.30 |
| Rhesus Monkey H2A.Z-2 | 30.50 | 28.12 | 24.22 | 11.72 | 14.06 | 10.94 | 7.03 | 77.90 | 38.30 |
| Chimpanzee H2A.Z-2 | 30.50 | 31.26 | 21.87 | 11.72 | 16.41 | 10.16 | 6.25 | 76.70 | 36.60 |
| Pig H2A.Z-2 | 44.00 | 28.12 | 24.22 | 11.72 | 14.06 | 10.94 | 7.03 | 77.90 | 40.00 |
| Cattle H2A.Z-2 | 33.80 | 28.12 | 24.22 | 11.72 | 14.06 | 10.94 | 7.03 | 77.90 | 40.00 |
| Sheep H2A.Z-2 | 33.80 | 28.12 | 24.22 | 10.94 | 14.84 | 10.94 | 7.03 | 77.90 | 40.00 |
| Dog H2A.Z-2 | 45.70 | 28.12 | 24.22 | 11.72 | 14.06 | 10.94 | 7.03 | 77.90 | 38.40 |
| Horse H2A.Z-2 | 31.90 | 28.58 | 22.85 | 11.43 | 14.29 | 10.00 | 6.43 | 76.50 | 37.50 |
| Mouse H2A.Z-2 (1) | 50.80 | 28.12 | 24.22 | 11.72 | 14.06 | 10.94 | 7.03 | 77.90 | 38.40 |
| Mouse H2A.Z-2 (2) | 50.80 | 28.12 | 24.22 | 11.72 | 14.06 | 10.94 | 7.03 | 77.90 | 38.40 |
| Rat H2A.Z-2 (1) | 38.90 | 28.12 | 24.22 | 11.72 | 14.06 | 10.94 | 7.03 | 77.90 | 45.00 |
| Rat H2A.Z-2 (2) | 38.90 | 28.91 | 24.22 | 10.94 | 14.06 | 10.16 | 5.47 | 76.00 | 46.60 |
| Rat H2A.Z-2 (3) | 38.90 | 28.12 | 24.22 | 11.72 | 14.06 | 10.94 | 7.03 | 77.90 | 45.00 |
| Rat H2A.Z-2 (4) | 38.90 | 28.91 | 24.22 | 10.94 | 14.06 | 10.16 | 5.47 | 76.00 | 46.60 |
| Rat H2A.Z-2 (5) | 38.90 | 28.90 | 25.00 | 11.72 | 14.84 | 10.16 | 7.03 | 77.90 | 46.70 |
| Rat H2A.Z-2 (6) | 38.90 | 28.90 | 25.00 | 11.72 | 14.84 | 10.16 | 7.03 | 77.90 | 46.70 |
| Rat H2A.Z-2 (7) | 38.90 | 28.12 | 24.22 | 11.72 | 14.06 | 10.94 | 7.03 | 77.90 | 45.00 |
| M. domestica H2A.Z-2 | 44.10 | 26.56 | 24.22 | 11.72 | 12.50 | 10.94 | 7.03 | 77.90 | 40.00 |
| Zebrafish H2A.Z-2 (1) | 27.20 | 28.12 | 24.22 | 11.72 | 14.06 | 10.94 | 7.03 | 77.90 | 50.00 |
| Zebrafish H2A.Z-2 (2) | 37.30 | 25.78 | 24.22 | 10.94 | 12.50 | 10.16 | 7.03 | 76.20 | 49.30 |
| X. tropicalis H2A.Z-2 | 32.20 | 28.12 | 24.22 | 11.72 | 14.06 | 10.94 | 7.03 | 77.90 | 36.60 |
| X. laevis H2A.Z-2 (1) | 35.60 | 28.12 | 24.22 | 11.72 | 14.06 | 10.94 | 7.03 | 77.90 | 38.30 |
| X. laevis H2A.Z-2 (2) | 33.90 | 28.12 | 24.22 | 11.72 | 14.06 | 10.94 | 7.03 | 75.00 | 51.60 |
| Average | 38.74±10.38 | 28.05±1.17 | 24.04±0.72 | 11.47±0.49 | 14.07±0.85 | 10.59±0.55 | 6.86±0.67 | 77.49±0.84 | 41.76±4.73 |

* See Additional file 2 for GenBank accession numbers.
